# Preparation and Characterization of Isostructural Na_2_MoO_4_ and Na_2_WO_4_ and a Study of the Composition of Their Mixed System

**DOI:** 10.3390/molecules28186602

**Published:** 2023-09-13

**Authors:** Alberto Ubaldini, Flavio Cicconi, Antonietta Rizzo, Stefano Salvi, Vincenzo Cuzzola, Francesco Gennerini, Stefania Bruni, Giuseppe Marghella, Alessandro Gessi, Naomi Falsini

**Affiliations:** 1 Italian National Agency for New Technologies, Energy and Sustainable Economic Development (ENEA), Via Martiri di Monte Sole 4, 40129 Bologna, Italy; antonietta.rizzo@enea.it (A.R.); stefania.bruni@enea.it (S.B.); giuseppe.marghella@enea.it (G.M.); alessandro.gessi@enea.it (A.G.); naomi.falsini@enea.it (N.F.); 2 Italian National Agency for New Technologies, Energy and Sustainable Economic Development (ENEA), C.R. Brasimone, 40032 Camugnano, Italy; flavio.cicconi@enea.it (F.C.); stefano.salvi@enea.it (S.S.); vincenzo.cuzzola@enea.it (V.C.); 3 Department of Electrical, Electronic and Information Engineering “Guglielmo Marconi” (DEI), Biomedical Engineering, Cesena Campus, Alma Mater Studiorum University of Bologna, Via dell’Università 50, 47522 Cesena, Italy; francesco.gennerini@studio.unibo.it

**Keywords:** Na_2_MoO_4_, Na_2_WO_4_, spectroscopic characterization, solid solutions, synthesis, material science

## Abstract

Na_2_MoO_4_ and Na_2_WO_4_ are isostructural semiconductors, belonging to the spinel class. They have interesting properties and find applications in numerous sectors. These properties can be tuned by controlling the composition of their solid solutions. Here, different methods to obtain these compounds are presented, both wet and solid-state synthesis. The obtained results show a possible dependence of the material properties on the chosen synthesis method. The pure compounds and their mixtures were characterized by Raman spectroscopy, scanning electron microscopy, and X-ray diffraction.

## 1. Introduction

A solid solution is a homogeneous solid phase, formed by a mixture of two or more different components with a defined microscopic structure that does not change as a consequence of the composition, at least for some range [1,2]. Generally, this microscopic structure is crystalline and, in this case, the different components that can be atoms, ions or molecules, randomly occupy some specific reticular positions of the crystalline structure, without changing its symmetry properties. The cell parameters can change as a function of the composition, but the crystalline cell remains the same. Specifically, the mixture of the different components does not lead to the formation of a new crystalline phase.

Solid solutions are a topic of interest for many fields of materials science, from metallurgy to geology, and there are examples in many different classes of materials: metals, ceramics, salts, and organic compounds [1,3].

The formation of a solid solution depends on the chemical, crystallographic properties, and often on quantum and electronic features of the parent substances. Substitutional solid solutions, according to the Hume–Rothery rules [4,5], can form if the solvent and the solute have the following criteria: similar atomic radii (15% or less difference), the same crystalline structure, similar electronegativity, the same or similar valence. Generally speaking, isostructural compounds, with isovalent elements, are good candidates for the formation of solid solutions.

In addition to the solid solutions in which the different components replace each other, in specific lattice positions, there are also the so-called interstitial solutions where small atoms can insert themselves into the free space between larger atoms. An example is the case of carbon atoms in steels [6,7]. Both of these types of solid solutions affect material properties by distorting the crystal lattice and perturbing the physical and electrical homogeneity [1,8]. In any case, it must be kept in mind that solid solutions can exist for any stoichiometric ratio between the components which, consequently, can vary from 0% to 100%, or in more restricted ranges, which can be near the extremes of compositions, i.e., close to pure parent compounds or ranges around specific compositions. In addition, all these solubility ranges depend on thermodynamic conditions, such as temperature and pressure.

Chemical, physical, mechanical, and optical properties of solid solutions, in general, change as a function of the composition, due to thermodynamic and quantum reasons, as different states and orbitals can be involved, as the number of valence electrons or of concentration or nature of charge carriers can change, and this can allow for an effective and specific tuning of these properties for many different technological applications [9,10].

Solid solutions can be often prepared by a direct reaction between the parent components according the general and formal reaction [1,2]:

(1 − x) AB + x CB → A_1−x_C_x_B

through chemical paths involving the liquid state by controlled fusion and solidification (something usually carried out by metallurgists for the preparation of steels and metallic alloys [11,12]), or through solid-state reactions at high temperature (as it is very often made for ceramic compounds [13,14]) or at high pressure [15].

However, in some cases, it is possible to prepare them using other “soft” methods at low temperature [16]. Among them, one of the most significant is the co-precipitation route [17,18]. Briefly, the principle of this method is to prepare liquid solutions of the parent compounds at low temperature, mix them in the desired ratios, and then add a suitable precipitating agent. In this way, if the precipitating agent is correct, i.e., if the product which is formed is very insoluble in that medium and the precipitation is complete, a phase is formed in which the elements of the solution are homogeneously mixed at atomic or molecular scales. The precipitated phase can be directly the one of interest or it can be an intermediate compound that can be transformed into the final product by means of low-temperature heat treatments. This method is often used in the preparation of oxides, carbonates, mixed halides or other similar compounds and, sometimes, for the preparation of nanoparticles and other nano/micro-objects [18]. Precipitating agents, such as oxalic acid, phosphoric acid, and hydrogen sulfide, are often very effective, because most of their transition metals and rare earth salts are highly insoluble; therefore, they allow for very rapid and complete precipitation [19,20]. A “good” precipitation is not only a kinetic problem, but the salts of these precipitating agents are very insoluble that the conditions for solid phase formation are achieved immediately, so that the homogeneous structure of the liquid is immediately “frozen” and it can be maintained in the precipitated particles. By using less suitable precipitating agents, the process can be slower and phenomena of segregation, local enrichment or phase separation can take place.

In this work, these general concepts have been applied to the case of Na_2_MoO_4_ and Na_2_WO_4_. The two pure compounds are isostructural, belonging to the spinel class, the space group of *Fd-3m*, have very close cell parameters, and are compounds of transition metals of the same group of the periodic table (VI) [21,22,23]. Thus, these compounds might be expected to have the best characteristics for being the end groups of a complete solid solution.

They are large gap semiconductors [22,24] and have attracted great interest because they have been used for many applications, such as in the preparation of coated electrodes for electrocatalysis [25], as additives for supercapacitor [26], as a fire retardant [27], as promising materials for optoelectronic applications [23,28], due to excellent dielectric constant, refractive index, absorption of light, reflection, and optical loss factor. In organic chemistry, sodium tungstate is used as a catalyst for epoxidation of alkenes and oxidation of alcohols into aldehydes or ketones and other reactions [29]. Their aqueous solutions are also very important because they are the starting point for the preparation of radiopharmaceuticals [30]. Molybdenum (^99^Mo) and tungsten (^188^W) are the parent isotopes for ^99^Tc [31] and ^188^Re [32], which are important for diagnostic and therapeutic nuclear medicine. Activated aqueous solutions of Na_2_MoO_4_ and Na_2_WO_4_ are used for feeding radioisotope generators, from which pertechnetates (TcO_4_^−^) and perrhenates (ReO_4_^−^) are extracted by elution [32,33].

For these reasons, the study of these pure compounds and their mixtures is of great interest. Some features, such as the band gap, optical properties, and catalytic aspects, may be tuned by the formation of a solid solution between them.

Molybdenum and tungsten have many similar chemical properties, including their reactivity with respect to concentrated H_2_O_2_ solutions [34]. This also suggests that a solid solution can form. The reaction between these metals and the hydrogen peroxide leads to the formation of molybdic acid (H_2_MoO_4_) and tungstic acid (H_2_WO_4_), respectively, from which increasing the pH makes it possible to obtain solutions of Na_2_MoO_4_ and Na_2_WO_4_. It is, in principle, rather easy to imagine a way to prepare solutions with a desiderated ratio between these two metals, which could be the starting point for solid solutions Na_2_Mo_1−x_W_x_O_4_.

They could be prepared by a classical solid-state method, which consists of making the precursors react at high temperature, or by the wet chemical method. Actually, both pure substances are insoluble in ethanol, which, consequently, could be used as a precipitating agent.

However, in general, the preparation and properties of these mixtures may depend on the route of synthesis and it is worth investigating the influence of the used methods, namely, if different chemical pathways lead to the same results. In this work, mixed Na_2_Mo_1−x_W_x_O_4_ samples were prepared using some co-precipitation methods and a more traditional solid-state synthesis for comparison. The solubility limits and the chemical–physical properties of these solutions have been investigated by means of scanning electron microscopy (SEM), Raman spectroscopy, X-ray diffraction.

## 2. Results and Discussion

**Figure 1:**
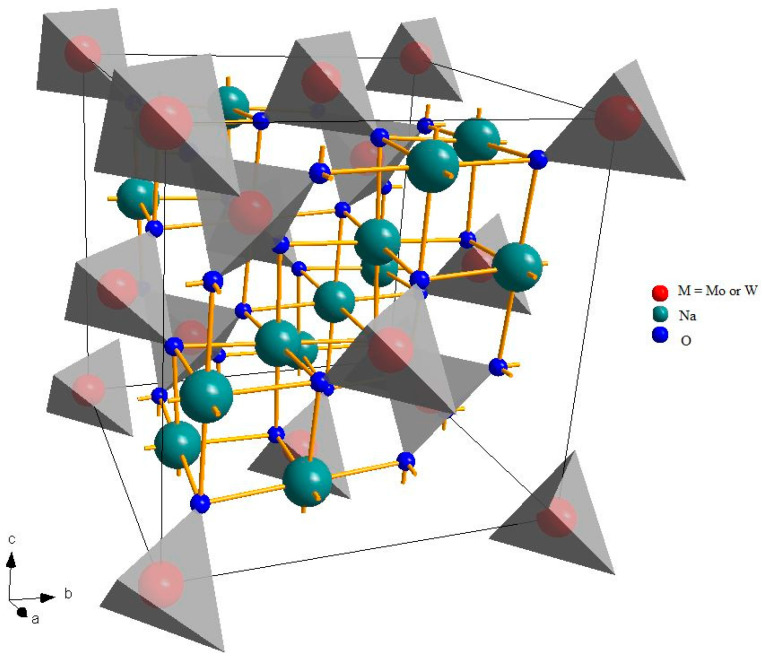
Crystalline structure of Na_2_Mo(W)O_4_. The gray tetrahedra, around the transition metal atoms, represent the local coordination of the BO_4_ units.

Figure 1 shows the crystalline structure of a spinel, a very wide group of materials with general formula A_2_BX_4_, where A and B are cations and X is an anion. The prototype material of this family is the oxide of magnesium and aluminum, MgAl_2_O_4_ [35].

Despite the fact that different symmetries are possible, spinels usually crystallize in the cubic crystal system and the A and B cations have octahedral and tetrahedral coordination, respectively (eight tetrahedral and four octahedral sites per formula unit) [36]. This structure is rather versatile and can accommodate different types of cations, with different charges. Na_2_MoO_4_ and Na_2_WO_4_ are a rare combination of monovalent and hexavalent cations. At room temperature, they are both cubic, even if at higher temperatures, there are some phase transitions to other structures. Tungsten is significantly heavier than molybdenum (atomic number 74 and atomic mass 183.84 a.u. against 42 and 95.95 a.u.) and belong to a lower periodic group, but they have a very similar ionic radius (in tetrahedral coordination and hexavalent state, 0.41 Å for molybdenum and 0.42 Å for tungsten [37,38]), leading to very similar cell parameters: about 9.11 Å for Na_2_MoO_4_ [21] and 9.13 Å for Na_2_WO_4_ [22]. It might be expected, therefore, that in a solid solution, the two cations could be hosted in the tetrahedral sites, with a random distribution, proportional to the composition.

In this work, pure compounds Na_2_MoO_4_ and Na_2_WO_4_ have been prepared, exploiting the chemical reactivity of the respective transition metals towards hydrogen peroxide, according to a method which has been proven to be very effective: the dissolution of the metals with the peroxide is rapid and complete, and the subsequent steps for the formation of solids do not present particular critical issues.

Formally, the overall reaction can be written as follows:

Mo(W)(s) + 3 H_2_O_2_ (l) + 2 NaOH (l) → Na_2_Mo(W)O_4_ (l) + 4 H_2_O

From these liquid solutions, it is easy to crystallize a solid, and any NaOH impurities can be removed by several washes with ethanol. Hydrogen peroxide under acidic pH is a good oxidant, according the semi reaction: H_2_O_2_ + 2H^+^ + 2e^−^ → 2H_2_O, so that many metals under these conditions are oxidized to higher valence states. Often, these ions are more soluble in aqueous media, and this can explain why there is a release from the metals to the solution.

Experimental observations have shown that molybdenum is more reactive than tungsten, i.e., with the same mass, volume of peroxide and other conditions, the former dissolves completely faster than the latter and, in both cases, the reaction initially is slow for a short induction time, which depends on the temperature. However, once the reaction starts, it proceeds rapidly with a large development of heat (they are highly exothermic) and the formation of a large quantity of bubbles, mainly oxygen [34].

This is possibly due to the fact that the surfaces of metallic pieces are covered by a very thin layer of oxide with intermediate valence, such as MoO_2_, and that it acts as a passivation layer. Only when this passivation layer is no longer complete and the underlying metal is exposed to the aggressive solution does the reaction become more rapid. Furthermore, the process leads to the formation of acid species which reduce the pH, making the peroxide itself more and more aggressive. In the case of molybdenum, the color of the solutions changes as the volume of peroxide increases, becoming pale yellow due to the formation of molybdic acid and other similar anions. This color variation is not seen in the case of tungsten, but the same occurs in the formation of tungstic acid. In these conditions, the process becomes autocatalytic and the reaction extremely fast.

**Figure 2:**
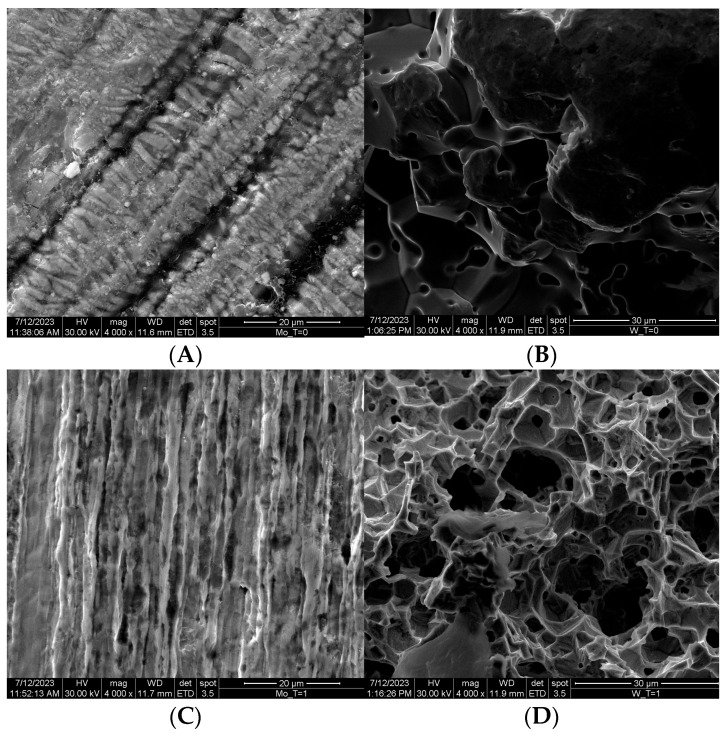
SEM images of the initial Mo (**A**) and W (**B**), before the reaction with the peroxide solution and after 15 min for Mo (**C**) and after 45 min for W (**D**).

At microscopic level, even in the early stages of the dissolution process, it is possible to notice that there are clear signs of corrosion on the surface of the metal pieces, like small craters, lines and other marks, which become larger over time. In general, after a sufficiently long time, these initial corrosion points become more deep and their morphology becomes quite reminiscent of pitting. Corroded areas and craters have different widths and depths, and often the wider ones are proportionately much deeper. These corrosion marks, especially the larger ones, are often separated by thin walls that meet at angles of about 120°. They are the areas from where hexavalent ions are released into the solution. Figure 2A–D shows the evolution of the corrosion of metals exposed to the peroxide solution as a function of time, from time 0 (upper line) and after a certain time (lower line) on the left for Mo and on the right for W.

Once the dissolution of the metals is complete and the pH is increased by NaOH addition up to a high value, it is possible to crystallize the materials, according to the procedure described in the experimental section.

**Figure 3:**
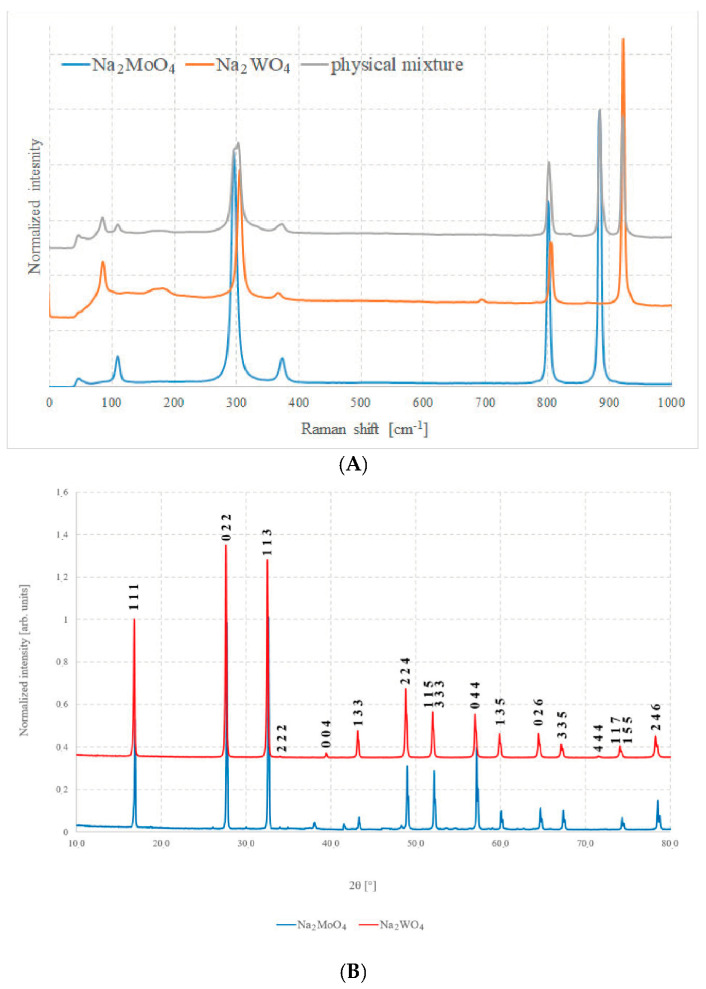
(**A**) Raman spectra of Na_2_MoO_4_, Na_2_WO_4_, and of a 50% mixture of the two and (**B**) XRD patterns of Na_2_MoO_4_ and Na_2_WO_4_.

Figure 3A shows the Raman spectra of the two pure compounds and Figure 3B shows the XRD patterns of the same samples. In Figure 3A, the spectrum of an equimolar physical mixture of pure Na_2_MoO_4_ and Na_2_WO_4_ can be observed, namely, a mixture in which the two components have been ground and mixed intimately, but without any type of chemical process between them.

Both techniques highlight the good quality of the samples prepared with the described method, as all peaks and bands can be unambiguously assigned to their respective phases, with just a very small impurity such as sodium carbonate, Na_2_CO_3_.

The crystalline structure consists of BO_4_ tetrahedra (B = Mo or W) and NaO_6_ octahedra; the crystals have eight formula units per unit cell [23]. In the crystal, B cations occupy Td sites, whereas Na ions occupy D3d sites. From a spectroscopic point of view, these tetrahedral sites can be seen as almost isolated units and their vibrational modes are the main features that can be observed in both cases. According to the standard group theory, the irreducible representation Γ admits 39 optical modes, which are as follows [21,22,28]:

Γ = A_1g_ + E_g_ + F_1_g + 3F_2g_ + 2A_2u_ + 2E_u_ + 4F_1u_ + 2F_2u_

Selection rules, anyway, allow the A_1g_, E_g_, and F_2g_ modes to be Raman active due to symmetric stretching (υ1), asymmetric stretching (υ3), symmetric bending (δ2), and asymmetric bending (δ4) of the MoO_4_ units. In the case of molybdenum compound, the measured bands match well with what has been reported: 891 cm^−1^, 808 cm^−1^, 380 cm^−1^, and 302 cm^−1^. In addition to these bands, it is possible to note a further signal at about 115 cm^−1^ that can be assigned to the vibrational collective mode of crystalline lattice [22]. For the Na_2_WO_4_, the bands have the same origin, but their position in the spectrum is different: 928 cm^−1^, 812 cm^−1^, 371 cm^−1^, and 312 cm^−1^, as well as a signal at about 90 cm^−1^.

Na_2_MoO_4_ has an additional signal at about 50 cm^−1^. It could have the same origin, i.e., be a second band due to other reticular motions, but being very close to the stimulating radiation, it cannot be excluded that it is an instrumental artifact. This peak will not be discussed further.

It is worth noting, in any case, that in both cases no signals are evident in the OH stretching region of the spectrum. Although Raman spectroscopy is not best suited to detect the presence of water or hydroxyl groups, this observation strongly suggests that the samples are anhydrous.

Assignments of the bands, obtained from Reference [22], are reported in Table 1.

Interestingly, these bands do not behave in the same way: three of those that are associated to BO_4_ intrinsic vibrations are at higher wavenumbers when B is tungsten, while the one at about 370 cm^−1^ moves in the opposite direction and is at higher wavenumbers for Na_2_MoO_4_. In addition, the shift is not the same for all the signals, being the largest one is for the symmetric stretching (υ1). The bands due to the lattice collective translational motions are at higher wavenumbers for Na_2_MoO_4_ than for Na_2_WO_4_. The observation that some modes are at higher wavenumbers for the second compound might appear surprising, because tungsten is heavier than molybdenum.

In a classical and simplified model, rigorously valid only for diatomic molecules, the vibrational frequencies of a bond between an A atom and a B atom with mass M_A_ and M_B_, are determined by the strength of the bond and the mass of the atoms involved in the vibration, according to the formula:

$$\nu \;=\;\frac{1}{2\pi }\sqrt{\frac{k}{\mu }}$$

where ν is the frequency, *k* is the strength constant of that bond, and *μ* is the reduced mass, i.e.,:

$$\mu =\frac{M_{A}M_{B}}{M_{A}+M_{B}}$$

For these compounds, only tetrahedra formed by oxygen atoms and Mo or W atoms are involved, so that *μ* is greater for the second one; therefore, it might be expected that the bands for this compound were at lower wavenumbers than those for Na_2_MoO_4_.

For instance, this is observed in the case of Raman spectrum of heavy water (D_2_O) [39], where deuterium atoms replace hydrogen atoms: all the bands are shifted towards lower wavenumbers, being the bond force the same, while the mass of deuterium is double. Thus, assuming that the bond strengths between metals and oxygen were the same, the bands in Na_2_WO_4_ should be all at lower wavenumbers than the corresponding bands in Na_2_MoO_4_. This is not the case, suggesting that the bonds between oxygen and tungsten are more rigid than those between oxygen and molybdenum. Considering that the distance among the atoms and the angles among them practically do not change, being cell parameters almost the same, this stiffening is not due to a reduction in the interatomic distances. A clue to a possible explanation of this phenomenon could be seen in the higher electronegativity of tungsten compared to that of molybdenum (2.36 against 2.16, respectively [40]), which could give a more ionic character to the bond Mo–O. Further investigations would be required to better understand this point. In any case, it seems that W–O bonding is somehow stronger than that with molybdenum. Indeed, since in general, the stretching vibrations are stiffer than the bending vibrations, this could explain why the bands that correspond to them move towards higher wavenumbers, while this effect is less evident for the others (even, one moves in the opposite direction): for the former group, the effect of the bond strength is the driving one, while for the latter ones, the mass variation is more important. Specifically, the higher mass of tungsten compared to molybdenum mass should move all the peaks towards a lower position and, since this does not happen, there must be an increase in the strength constant of the bonds W–O that are able to compensate and overcome this. Vibrations involving atomic positions (stretching) are stiffer than those of bond angles (bending). Therefore, for the former group, the effect due to the constant strength should be greater.

Some additional considerations can be carried out in regards to the low wavenumber part of the spectra of these compounds. Generally speaking, in solids, the part of the spectra below 150–200 cm^−1^ corresponds to collective vibrations: lattice vibrations and or intermolecular modes. In particular, the lattice modes are either translational motions of the center of mass of the molecules or hindered molecular rotation of blocks or structural sub-units, reflecting the short-range order [41]. These modes are sensitive to the size of the crystallites, to the presence of some amorphous components or to other structural defects.

All of the bands in a Raman spectrum of a solid arise from phonons, and some crystal lattice vibrational modes can be thought of as the collective motion of molecules or of some crystalline subunits as a whole. For example, in layered materials, such as transition metal chalcogenides, boron nitride, black phosphorus and others, some low-frequency Raman bands are a result of the vibrations of entire layers, and either perpendicular (shear) modes or parallel, (breathing) modes are possible [42]. The shear modes can be pictured as atomic or molecular layers moving antiparallel to each other within their respective planes, whereas the breathing modes involve the layers moving away from and towards each other [42,43]. In general, in the majority of crystalline materials, there are bands that are the result of the vibration of some large blocks or subunits instead of being the vibration of some specific atoms, as expected by the irreducible representation. They are generally of low energy and absent in the liquid or gas spectrum of the same material because they lack a long-range translational symmetry that is typical of a crystal. Considering that the distance among the vibrating elements is large, the force among them is normally weak; therefore, in most cases, the reduced mass of the oscillator is the most important parameter, and these bands shift as a function of the reduced mass [44,45].

This is the case for bands at the lowest wavenumbers in both spectra shown here, that have been assigned to the collective lattice vibrations of BO_4_ tetrahedra. In agreement with these considerations, the signal is at a much higher position in wavenumbers for Na_2_MoO_4_ than for Na_2_WO_4_.

In any case, these Raman spectra demonstrate the good quality, the high purity, and the good degree of crystallinity of the materials thus prepared, due to the absence of spurious peaks and because all the bands are extremely sharp and rather narrow.

Similar considerations can be carried out for XRD analysis. It is possible to recognize the high purity and excellent quality of these samples since all the peaks of the diffractogram can be almost uniquely attributed only to the pure compounds, and just very small quantities of other oxides are present in the case of Na_2_MoO_4_. The peaks do not shift significantly, because the cells have almost the same volume and atomic spacing. This, unfortunately, leads to the exclusion of diffraction analysis as the main mean of investigation for solid, because it is difficult to attribute any change in position to a compositional variation.

Actually, it can be expected that the XRD peaks in a generic solid solution A_1−x_C_x_B move as a function of x, due to the changes in cell parameters and atomic spacing. However, in this specific case, it is difficult to measure any variation in the peak positions, since they are already at very similar angles in the pure compounds.

From a spectroscopic point of view, when a solid solution is analyzed, something similar can also be observed. The spectroscopic analysis could be more complex if fluorescence was present or if the Raman response was much stronger for one of the two compounds, but also in this case, a shift of the bands as a function of the composition can be expected. However, there is another possibility: when the mutual substitution is not completely random, but some kind of long-range ordering takes place, due to a change in symmetry or to the formation of a superstructure, new bands, in addition to those already existing, can appear.

The physically mixed sample can be imagined as a reference. In this case, precisely because no chemical reaction has taken place between the two compounds, but they have just simply been mixed, all the bands of both oxides are present, in the expected position and with an intensity proportional to their quantity. Zero solubility of W in Na_2_MoO_4_ and of Mo in Na_2_WO_4_ would result in spectra similar to this one: the bands would be those of the two pure systems, with intensities proportional to the relative concentrations without any change in position. Conversely, if the reciprocal solubilities are not zero, a shift of these bands could be expected. Finally, if there is a maximum solubility limit for the reciprocal substitutions, i.e., if there is a miscibility gap, the shift of the bands would occur only up to a certain composition, beyond which, there would be no further shifts, but only variations in the relative intensities.

In the present work, the mixtures of Na_2_MoO_4_ and Na_2_WO_4_ were prepared according to three different methods, described in detail in the following section, in terms of the experimental methods and called A, B (B1 and B2), and C.

The first consists of preparing the mixtures with the desired ratio, of pure transition metals, because the stoichiometric ratio is equal to one. In this case, the same considerations on reactivity, described previously, apply and the first solution is obtained by adding hydrogen peroxide. Subsequently, when the last metallic particle completely disappears, NaOH is added in excess. The other methods, on the other hand, start from pure compounds, mixed in the correct ratio, in aqueous solution (B) exploiting their solubility in water, or in the solid state (C).

Surprisingly, the spectroscopic characterization of the mixtures prepared by all these methods has shown that the formation of a complete solid solution for any value of x does not occur. On the contrary, the relative solubilities, i.e., the highest solubility of W in Na_2_MoO_4_ and of Mo in Na_2_WO_4_, are very modest.

**Figure 4:**
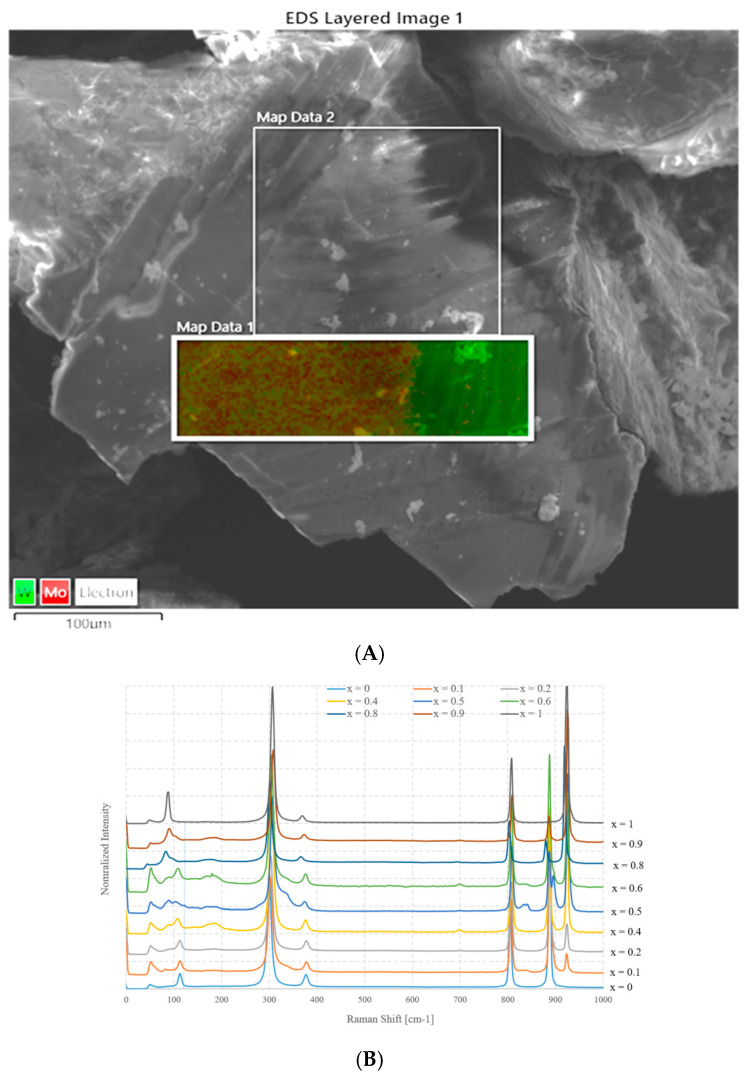
(**A**) SEM image and EDX chart distribution of Mo and W in Na_2_Mo_0.5_W_0.5_O_4_ sample, (**B**) Raman spectra as a function of x (values from 0 to 1) for samples prepared according to method A.

Figure 4A shows an SEM image and the EDX element distribution charts of a sample with nominal composition Na_2_Mo_0.5_W_0.5_O_4_ prepared using method A. Figure 4B instead shows the Raman spectra as a function of x.

The first figure puts in evidence that a separation of phases occurs, because, while the average composition, measured over a large area, is very close to the nominal one (Na_2_Mo_0.47_W_0.53_O_4_), this sample is formed by two well distinct areas, and where tungsten is abundant, molybdenum is almost absent. On the contrary, it seems that in other parts of the sample, there is a limited coexistence of the two elements, although the molybdenum content is much larger. This suggests that nearly no substitution of W by Mo occurs in Na_2_WO_4_, whereas it is possible that a partial replacement occurs in the other case, i.e., Mo by W occurs in Na_2_MoO_4_. The composition of the molybdenum rich side is close to Na_2_Mo_0.72_W_0.28_O_4_.

The spectra of the mixed samples are more complex than those of the parent compounds and a higher number of rather narrow bands is recognizable, although some of these peaks are due to small residual amounts of NaOH. However, the main bands can easily be referred to those of pure Na_2_MoO_4_ or Na_2_WO_4_.

Some observations can be made: the first is that the intensity of these bands changes as a function of x and those which are due to Na_2_WO_4_ increase as it grows, while the others behave in the opposite way. In general, the bands do not shift significantly with respect to the positions of their respective parent compounds, at least in the limit of spectral resolution.

While in the case of the bands that are due to Na_2_WO_4_, this is not surprising and in good agreement with the SEM–EDS analysis, because there is no chemical substitution, some shifts could be expected for the bands referred to as Na_2_MoO_4_, even if very small.

Considering, for example, the mode corresponding to symmetric stretching, the difference for the two pure compounds is about 35 cm^−1^. Assuming a linear shift of this band as a function of composition, a change in composition equal to x = 0.1 should lead to a shift of about 3 cm^−1^ (for the other bands, however, it would be much lower than this value). Thus, for x = 0.3, the band should move from about 980 cm^−1^ to about 990 cm^−1^. The possibility that the two phases are still spatially separated at a smaller scale, i.e., at submicrometric scale or even nanometric, without a real chemical substitution cannot be ruled out. On the contrary, it seems very realistic.

The part of the spectra below 120 cm^−1^ is interesting. While the band of the tungsten compound is always recognizable and its intensity increases with x, the band due to the collective motions in the Na_2_MoO_4_ is more sensitive to doping. Its intensity decreases with the concentration of tungsten and, at the same time, it shifts towards lower wavenumbers as a function of x, at least for x < 0.3. Thus, the presence of tungsten in any case disturbs the long-range collective reticular modes. This could be due both to a modest level of chemical substitution and to the reduction in local symmetry on the nanoscale.

Thus, it can be stated that these samples prepared by method A are not a single phase but they are biphasic. One of the two phases present is certainly pure Na_2_WO_4_, without any Mo doping, while the other one is Na_2_MoO_4_. In this case, it is possible that a small substitution takes place, but in any case, very modest.

Method A consists of evaporating quickly the water from the solution, leading to the formation of a solid residue. These two oxides are both quite soluble in water and the solid residue appears when most of the liquid is already evaporated. This could justify the result of phase segregation. Since the solubility cannot be exactly the same for the two compounds, being Na_2_WO_4_ slightly more soluble, a process similar to a fractional crystallization can take place. Even if this phenomenon cannot be considered a process at thermodynamic equilibrium, as the solvent evaporates, saturation conditions are quickly achieved and the more the liquid evaporates, the more the crystalline solid forms.

During a fractional crystallization process, the first precipitate will contain more of the least soluble substance, in this case Na_2_WO_4_, while the residual liquid becomes richer and richer in the other, i.e., tungsten. In the first crystals, a certain amount of tungsten can still be incorporated, although not uniformly throughout, while almost pure sodium tungstate particles are eventually formed, because by the time they appear, all the molybdenum has already been consumed.

**Figure 5:**
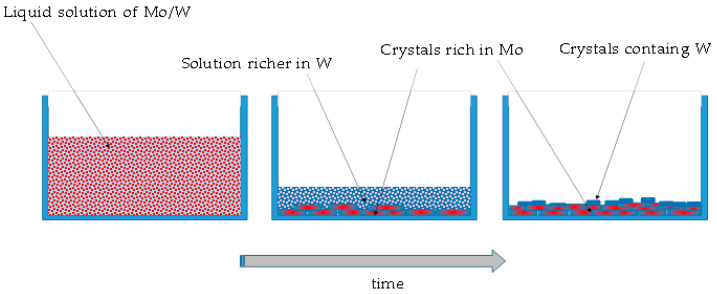
A schematic representation of formation of the solid residue using method A.

The phase separation, in this case, is therefore due to purely thermodynamic reasons and parameters, such as the evaporation rate, the process temperature, or the initial concentration can play a role leading to small differences in final product in terms of size of the particles and of the presence of compositional gradients. Figure 5 shows a schematic representation of this process.

**Figure 6:**
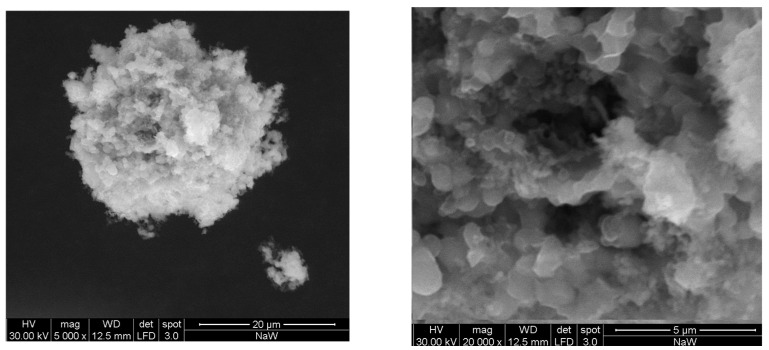
SEM images, at different magnifications, of a sample Na_2_WO_4_, prepared according to method B2.

For this reason, the other synthesis methods have been developed, to try to force substitution at the crystalline level. In contrast to the first route, method B involves the use of a precipitating agent, in this case ethanol, in which both substances are insoluble. The idea is that, in this way, it is possible to maintain the homogeneous distribution of the liquid solution, thanks to an out-of-equilibrium process. In method B1, it is added dropwise to the solution, in method B2 instead, it is the aqueous solution which is added to a large excess of ethanol. In both cases, critical conditions for precipitation are quickly achieved, at least locally. In the second case, small particles form almost immediately and become growth nuclei for the precipitate, whereas the in the former one, it takes some time before the solution becomes turbid and a precipitate forms, likely because an initial redissolution occurs. The particles which are formed are rather small as it can be observed in Figure 6, which shows some particles of Na_2_WO_4_, prepared according to method B2.

It is possible to see aggregates with a roughly round shape of a few microns in diameter (on the left). These aggregates are made up of many much smaller crystalline particles (on the right). These particles are flat and have an average size of less than a micron, but there are many even smaller.

In regards to the compositions, no particular differences are observed with respect to the previous case. Even for x = 0.1 and x = 0.9, the system is biphasic because the bands due to the secondary phase are very weak, but still evident. For any intermediate composition, the Raman spectra show coexistence of the bands of the two compounds with intensities proportional to their relative quantities. This strongly indicates that the non-formation of the complete solid solution is not due only to the method of preparation.

**Figure 7:**
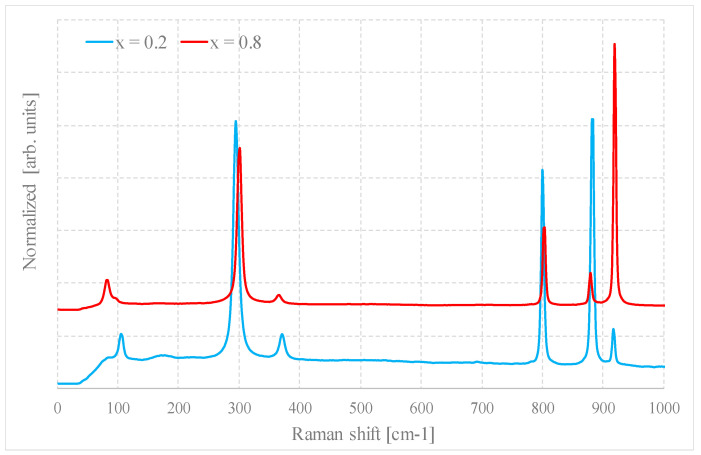
Raman spectra of samples with x = 0.2 and 0.8 prepared by method B1.

Figure 7 shows as an example the Raman spectra of a sample for x = 0.2 and x = 0.8, prepared by method B1.

The last preparation method studied in this work is the direct solid-state synthesis between mixtures of the precursors with the desired ratio (method C). Solid-state reactions are classical, bulk synthetic methods for fabricating inorganic solid materials, which consist of heating the starting materials mixed together to a high temperature. In this way, the reaction rates are largely increased and the diffusion of elements to their corresponding lattice positions is allowed; therefore, any local separation phenomena at the submicroscopic scale could be eliminated.

It has been reported that pure Na_2_WO_4_ was prepared by the solid-state reaction from a sodium carbonate and tungsten trioxide at 450 ° and then sintered at 650 °C, and pellets of Na_2_MoO_4_ were sintered in the temperature range of 600–800 °C; therefore, the temperature of 600 °C was considered adequate for this purpose. The mixed samples of Na_2_MoO_4_ and Na_2_WO_4_ in the desired ratios were held at this temperature in alumina crucibles for 24 h and then allowed to cool slowly.

At the end of this process, there was only a very slight loss of weight, of the order of 0.045%, at maximum, probably due to the very small evaporation of molybdenum and tungsten oxides, likely MoO_3_ and WO_3_. Under these conditions, a partial incipient melting of the samples was observed, which was also partially attached to the crucibles; however, it was possible to recover them and obtain the spectra in this case.

Again, no solid solution is formed and the spectra look very similar to what was shown above, except for some fluorescence which could be due to partial contamination, possibly due to an incipient reaction with the alumina. The bands appear to be quite narrow, but there is no visible shift.

While it is possible that much higher reaction times could have some effect, this result highlights that the non-formation of a single phase is not due to the method of preparation, but rather is an intrinsic feature of this system and evidently, it is not possible to induce a mutual chemical substitution between molybdenum and tungsten in this type of compound, at least using this synthesis method.

This result is in many ways very surprising because the parent materials are very similar and satisfy many of the general conditions that normally lead to the formation of solid solutions. Somehow, the chemical structure of these compounds, where the transition metals are within relatively isolated BO_4_ tetrahedra, is less susceptible to changes. It should be remembered that many other molybdates and tungstates exist, but few are composed of monovalent cations and have the spinel structure.

This could indicate that, in some cases, also other requirements have to be satisfied for their formation. For example, molybdenum and tungsten belong to the same group, but have different electron configurations, [Kr] 4d^5^ 5s^1^ and [Xe] 6s^2^ 4f^14^ 5d^4^, respectively. In the hexavalent state, tungsten has a complete f-shell and this could play a role in the determination of the HOMO and LUMO orbitals, and thus in the formation of some specific bonds. Slightly different oxygen contents in the pure phases are possible leading to a change in the average valence state of Mo or W and different local coordination.

This is not necessarily the explanation for single-phase formation failure in intermediate mixtures; however, it does indicate that in general, the considerations that must be made to predict the formation of solid solutions can be very complex.

## 3. Experimental Sections

High-purity molybdenum and tungsten were used in the form of pellets, supplied by Luoyang Combat Tungsten & Molybdenum Material Co., Ltd. (Luoyang, China) for the former one, and by Analysensysteme GmbH for the second one. Mo pellets are shaped similar to small cylinders a few hundred microns long and wide, while the W ones are just a little smaller with a more irregular shape. All pieces are very similar and just small variations in volume and weight can be observed. The purity of the samples has been analyzed by means of ICP—Triple Quadrupole—Mass spectrometer.

Their reaction with concentrated hydrogen peroxide solutions (at least 30% *w*/*w*) leads to their complete dissolution and to the formation of clear liquid. Different hydrogen peroxide solutions ranging from 30 to 40% *w*/*w* were obtained from Carlo Erba Reagents, Titolchimica and Sigma Aldrich. Highly concentrated hydrogen peroxide is not very stable and tends to decompose over time. For this reason, fresh batches were used as often as possible and, in general, it was kept in the freezer at −18 °C.

It is possible to obtain pure Na_2_MoO_4_ and Na_2_WO_4_ by increasing the pH through careful and slow addition of NaOH 1M (PanReac AppliChem Itw Reagents, Chicago, IL, USA) until the formation of completely colorless solutions, generally around pH 13. From them, it is possible to obtain the corresponding solid precipitates by adding ethanol. About 0.005 mol of pure metals, corresponding to about 0.48 g of Mo and to about 0.92 g of W, were used to prepare these oxides. According to the overall reaction leading to the formation of Na_2_MoO_4_ and Na_2_WO_4_, as seen in the discussion section, for every mole of metals three moles of H_2_O_2_ are required to achieve dissolution, and this means, making simple stoichiometric calculations, that for each gram of Mo or W about 3 mL and 1.7 mL of H_2_O_2_ (30% *w*/*w*), whose density is 1.11 g/cm^3^, are necessary respectively. However, taking into consideration the self-decomposition of peroxide and to make the reaction faster, at least three times this quantity was used in every experiment. After the dissolution, NaOH 1M was added slowly and carefully up to pH that is roughly equal to 13/14 and, in any case, until the solution was completely colorless. Again, for one mole of metals, two moles of hydroxide are required, and this means that for 1 gr of molybdenum about 21 mL of NaOH 1M and for 1 gr of W about 11 mL, are required respectively, but again a larger volume is necessary due to the excess of peroxide. At the stage when the pH is raised, the color of liquid changes several times and there is a strong formation of bubbles due to the decomposition of the excess of peroxides present. For this reason, it is better to add the basic solution slowly and not all at once, to avoid an uncontrolled process and so that the liquid does not escape from the container. At this point, it is possible to obtain the oxides either by drying the solution or by adding ethanol, which acts as a precipitating agent. The products were subsequently re-dissolved in water and re-precipitated several times with the same procedure in order to improve their purity and the NaOH excess is easily washed away.

Concerning the (1 − x) Na_2_MoO_4_/x Na_2_WO_4_ mixtures with different x ranging from 0 to 1, different synthesis approaches were used. The first, hereinafter referred to as method A, is to mix the metallic particles directly in the desired ratios, dissolve them completely by adding peroxide, and obtain a liquid solution by gradually increasing the pH by adding NaOH. At this point, a solid mixture is prepared by evaporating the solvent; the excess of hydroxide can be removed with several washes with ethanol, in which it is sufficiently soluble. The second approach was to mix the pure parent compounds in the desired quantities, obtain an aqueous solution, and at this point, it is possible to obtain the precipitation by adding ethanol dropwise (method B1) or by slowly dropping these solutions into an ethanol beaker (method B2). Finally, the last method (method C) was to make a solid-state reaction between the pure parent compounds in the desired ratios at 600 °C for 24 h.

In any case, the calculations were made in order to obtain 0.5 gr of the wished composition by changing the ratio between Mo and W or Na_2_MoO_4_ and Na_2_WO_4_, in an appropriate way.

**Figure 8:**
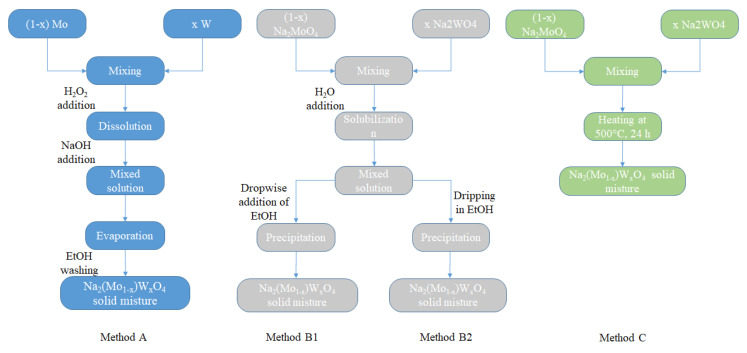
Flowchart of the preparation methods for Na_2_Mo_1−x_W_x_O_4_ mixtures.

Figure 8 shows a flowchart of these different methods of preparation.

In the present work, Raman spectra of the compounds and mixture were acquired, at room temperature, by a BWTEK i-Raman plus spectrometer equipped with a 785 nm laser to stimulate Raman scattering, which is measured in the range of 20–3500 cm^−1^ with a spectral resolution of 2 cm^−1^. The measurement parameters, acquisition time, number of repetitions, and laser energy, have been selected for each sample in order to maximize the signal-to-noise ratio.

X-ray powder diffraction (XRPD) investigations were performed in order to determine the crystalline phases, using a Philips X’Pert PRO 3040/60 diffractometer, operating at 40 kV, 40 mA, Bragg–Brentano geometry, equipped with a Cu Kα source (1.54178 Å) Ni filtered, and a curved graphite monochromator. PANalytical High Score software (version 4.1) was utilized for data elaboration.

The characterization, morphology, and composition of the samples have been performed by scanning electron microscopy (SEM-FEI Inspect-S) coupled with energy dispersive X-ray spectroscopy (Oxford Xplore).

## 4. Conclusions

Pure samples of Na_2_MoO_4_ and Na_2_WO_4_ have been prepared using an effective method, starting from the metals, by exploiting their reactivity with concentrated hydrogen peroxide. They are rapidly dissolved by the peroxide, and solutions of Na_2_MoO_4_ and Na_2_WO_4_ are formed by controlling the pH, which is increased by additions of NaOH. From these solutions, pure solid compounds are easily obtained. The samples prepared in this way have excellent quality, and they have been chemically and physically characterized. A discussion of the main features of their Raman spectra is presented. Interestingly, not all the bands behave in the same way, and some of them are at higher wavenumbers for the compound with W, while for others the opposite is observed.

The pure compound can be the parent for the formation of intermediate mixtures of Na_2_Mo_1−x_W_x_O_4_ with x ranging from 0 to 1. Different routes were followed for the preparation of the samples, at low temperature, by wet processes with or without the use of a precipitating agent (methods A and B) or, at high temperature, by a solid-state reaction (method C).

The chemical and crystallographic analogies between these elements and compounds could have predicted the formation of a single phase for any composition, instead what has been found, is that if any chemical substitution exists, it occurs for a composition very close to the pure end terms, whereas for any other intermediate composition, the system is biphasic.

Raman spectroscopy and SEM–EDS analysis have shown that there is no mutual replacement between Mo and W. This finding is dependent on the preparation method that has been used; therefore, it is an intrinsic feature of this system.

## Figures and Tables

**Table 1:** Assignments of the Raman bands [22].

Wavenumber (cm^−1^)				Attribution
Na_2_MoO_4_		Na_2_WO_4_		
Theoretical	Measured	Theoretical	Measured	
115	113	96	91	Collective Mode of (BO_4_)
302	300	312	309	δ_as_ (B–O)
380	377	371	372	δ_s_ (B–O)
808	806	812	810	ν_as_ (B–O)
891	888	928	925	ν_s_ (B–O)

## Data Availability

Not applicable.

## Sample Availability

Samples prepared during this study are available or they can prepared again following the same procedures.
